# Supplementary material for: Risk of Seizure Aggravation after COVID-19 Vaccinations in Patients with Epilepsy
Source: Vaccines (Basel). 2024 May 30;12(6):593. doi: 10.3390/vaccines12060593 (PMC11209536; doi:10.3390/vaccines12060593)
Supplement: Supplementary file 1 [file vaccines-12-00593-s001.zip › [COVACE] Supplementary Material S1 - Questionnaire.pdf]

Supplementary Material 1 – Structured Questionnaire

Verbal consent obtained: No / Yes / Carer (patient not fit for consent)
Questionnaire filled by: Patient / Carer

COVID-19 Infection: Yes / No
Rapid antigen test (RAT) positive date: ______________________
Polymerase chain reaction (PCR) test positive date: ______________________

COVID-19 Vaccination:

- ☐ First dose
  Date:
  Type of vaccine: *BNT162b2* (BioNTech) / *CoronaVac* (Sinovac) / Others: ______________________
  Number of seizures 7 days before first dose vaccination: ______________________
  Number of seizures 7 days after first dose vaccination: ______________________
  Number of seizures 30 days after first dose vaccination: ______________________
- ☐ Second dose
  Date:
  Type of vaccine: *BNT162b2* (BioNTech) / *CoronaVac* (Sinovac) / Others: ______________________
  Number of seizures 7 days before second dose vaccination: ______________________
  Number of seizures 7 days after second dose vaccination: ______________________
  Number of seizures 30 days after second dose vaccination: ______________________

Monthly Seizure Frequency (number of seizures per month):

Nov 2020: minor seizures: ______________________
major seizures: ______________________
seizures requiring hospitalization: ______________________

Dec 2020: minor seizures: ______________________
major seizures: ______________________
seizures requiring hospitalization: ______________________

Jan 2021: minor seizures: ______________________
major seizures: ______________________
seizures requiring hospitalization: ______________________

Feb 2021: minor seizures: ______________________
major seizures: ______________________
seizures requiring hospitalization: ______________________

Mar 2021: minor seizures: ______________________
major seizures: ______________________
seizures requiring hospitalization: ______________________

Apr 2021: minor seizures: ______________________
major seizures: ______________________
seizures requiring hospitalization: ______________________

May 2021: minor seizures: ______________________
major seizures: ______________________
seizures requiring hospitalization: ______________________

Jun 2021: minor seizures: ______________________
major seizures: ______________________
seizures requiring hospitalization: ______________________

Jul 2021: minor seizures: ______________________
major seizures: ______________________
seizures requiring hospitalization: ______________________

Aug 2021: minor seizures: ______________________
major seizures: ______________________
seizures requiring hospitalization: ______________________

Sep 2021: minor seizures: ______________________
major seizures: ______________________
seizures requiring hospitalization: ______________________

Oct 2021: minor seizures: ______________________
major seizures: ______________________
seizures requiring hospitalization: ______________________

Nov 2021: minor seizures: ______________________
major seizures: ______________________
seizures requiring hospitalization: ______________________

Dec 2021: minor seizures: ______________________
major seizures: ______________________
seizures requiring hospitalization: ______________________

Jan 2022: minor seizures: ______________________
major seizures: ______________________
seizures requiring hospitalization: ______________________

Feb 2022: minor seizures: ______________________
major seizures: ______________________
seizures requiring hospitalization: ______________________

Attitude towards vaccination:
Carer vaccinated instead of patient: Yes / No
Cues against vaccination (if unvaccinated):

- ☐ Fear of aggravating epilepsy
- ☐ Fear of unknown side effects
- ☐ Other chronic diseases
- ☐ Discouragement from healthcare providers
- ☐ Discouragement from media / social media
- ☐ Perceived lack of scientific evidence
- ☐ Low prevalence of COVID in HK
- ☐ Perceived low severity of COVID
- ☐ Carer vaccinated instead of patient
- ☐ Others (please specify): ______________________

Cues for vaccination (if vaccinated):

- ☐ Protection from infection
- ☐ Protect others from infection
- ☐ Safety of vaccine
- ☐ Encouragement from healthcare providers
- ☐ Encouragement from media / social media
- ☐ Social infective control measures
- ☐ Work / employment requirements
- ☐ Others (please specify): ______________________

Adverse effects after vaccination:

General adverse effects:

- ☐ Local side effects
- ☐ Fever
- ☐ Chills
- ☐ Fatigue
- ☐ Headache
- ☐ Myalgia
- ☐ Arthralgia
- ☐ Lymphadenopathy
- ☐ Sore throat
- ☐ Nausea / vomiting
- ☐ Diarrhea
- ☐ Others (please specify): ______________________

Epilepsy-related adverse effects:

- ☐ Subjective increase in minor seizure attacks
- ☐ Subjective increase in major seizure attacks
